# HERAS: A Modular Matlab Tool Using Physical Optics for the Analysis of Reflector Antennas

**DOI:** 10.3390/s23031425

**Published:** 2023-01-27

**Authors:** Alejandro Baldominos, Salvador Mercader-Pellicer, George Goussetis, Alberto Mengali, Nelson J. G. Fonseca

**Affiliations:** 1 Institute of Sensors Signals and Systems, Heriot-Watt University, Edinburgh EH14 4AS, UK; 2 Antenna and Sub-Millimetre Waves Section, European Space Agency, 2201 AZ Noordwijk, The Netherlands; 3 Telecom Systems and Techniques Section, European Space Agency, 2201 AZ Noordwijk, The Netherlands

**Keywords:** physical optics, reflector antennas, object-oriented programming, multi-beam antennas

## Abstract

HERAS is a tool developed in Matlab for the analysis of reflector antennas using physical optics (PO) theory. Its graphical user interface (GUI) and source code are freely available for educational and research purposes. It has the necessity of being a flexible tool to provide adaptability to system engineering requirements and can also be of interest to antenna engineers working on the design of reflector antennas. Due to the increasing demand of broadband services, satellite communications systems are becoming highly complex in order to meet connectivity requirements. To fully exploit the benefits of these systems, multidimensional optimisations are crucial, which call for an efficient estimation of the coverage characteristics. Reflector-based solutions are one of the preferred architectures for very high-throughput satellite (VHTS) systems. A detailed description of HERAS is presented in this paper which has been validated with available commercial software packages. In addition, some examples are described in which the tool has been used for the efficient estimation of VHTS systems performance.

## 1. Introduction

Satcom systems are increasingly moving from broadcast to broadband services, adapting to fast evolving consumer needs for high-throughput connectivity (e.g., video on demand). This marks the shift from broad-shaped beams providing regional or continental coverage to multiple spot-beam antenna architectures enabling spectrum reuse with a cellular-like coverage [1,2,3,4,5]. For a given frequency allocation, this approach enables a significant capacity increase. This transition is also accompanied by a shift to higher frequencies (e.g., moving from C-band to Ku and Ka-bands and above), thereby enabling wider untapped bandwidths. These systems are generally referred to as high-throughput satellites (HTSs), which provide a total capacity in the order of hundreds of Gbps. Multi-beam reflector antennas (MBRA) are one of the preferred configurations to achieve the requirements from geostationary orbit (GEO) due to the high gain offered by the system optics. Compared to direct radiating arrays (DRAs), MBRAs offer a favourable compromise between coverage characteristics and demands on the payload resources. Existing HTS systems implement passive MBRA architectures (e.g., ViaSat-1 [6], Eutelsat’s Ka-Sat [7], Jupiter-1 [8] etc.). These systems combine a reflector with a set of feeds located at the focal plane of the reflector, which are exploited to generate the multi-beam coverage. Depending on the number of feeds employed to generate each beam, two different configurations can be defined: single feed per beam (SFPB), where each beam is generated with one feed; and multiple feed per beam (MFPB), where several feeds generate each beam with the use of beam-forming networks (BFNs), providing adequate beam overlap. The trade-off between these two architectures relies on the coverage characteristics, the size of the on-board antenna farm, and the feed network complexity [5,9].

As we move towards the next generation of very high-throughput satellite (VHTS) systems, which target total throughput in the order of 1 Tbps, the number of beams is likely to grow from hundreds to thousands in order to achieve significantly larger spectrum reuse factors compared to existing HTS systems. This requires a careful design of the antenna system to provide a high carrier-to-interferer ratio (C/I), which has a direct impact on achievable data rates per user. Moreover, flexibility in the coverage characteristics, to account for short, mid and longer-term variations of the traffic demands, is becoming of increasing importance for optimising the satellite’s “sellable” capacity [10]. In order to meet these requirements, upcoming software-defined satellites mostly employ array-fed reflectors (AFR) in imaging configurations, where the reflector magnifies the gain produced by a primary feed array. AFR antenna architectures combine the high gain offered by reflector-based antenna solutions with the flexibility of active array solutions [11].

In order to maximise the benefits from the flexibility offered by the emerging payload architectures and optimise the system performance, VHTS systems are likely to also deploy more advanced resource (e.g., power, time, frequency, polarisation) allocation policies and interference mitigation techniques (e.g., user scheduling and/or precoding) [12]. Consequently, these systems are becoming increasingly complex, such that optimising and evaluating their performance becomes a multidimensional problem that depends not only on the antenna performance but also on the system architecture and operation, as well as the traffic characteristics, thus benefiting from a model-based design (MBD) approach.

When it comes to the antenna development, unlike heritage broadcast missions where shaped antennas could be designed against effective isotropic radiated power (EIRP) and gain-to-noise-temperature (G/T) contours, MBRAs for multi-beam and VHTS missions should thus be considered in the context of end-to-end system performance [13]. The co-design of the antenna with the payload becomes critical to optimise the performance of the satellite. In this context, estimating the multi-beam coverage characteristics becomes a step towards the desired system performance evaluation, which in turn is iteratively performed to optimise not only the antenna architecture but also the resource allocation and interference mitigation strategies. This calls for iterative estimations of the coverage characteristics by reflector antennas with several thousands of beams. The high computational demands associated with the aforementioned optimisation drives the need for efficient methodologies to obtain key characteristics of multiple beam coverage associated with the antenna subsystem. In order to accelerate the coverage estimation, system studies have so far relied on simple models for the antenna patterns based on closed form expressions [14], which are accurate enough for DRA architectures but do not sufficiently capture the beam degradation due to optical aberrations in reflector-based solutions, particularly for beams towards the edge of coverage. A more accurate evaluation of the antenna performance in trade-off with the computational effort is critical.

Traditional methods for the analysis of reflector antennas usually rely on numerical integrations, which are the main contributor to the total computation time. Physical optics (PO) is the preferred technique to predict the scattered field of electrically large structures such as reflector antennas, radar cross section of targets, etc. [15]. GRASP from TICRA [16] is the reference commercial tool for the analysis and design of reflector antennas, which uses a combination of PO and MoM techniques in order to analyse electrically smaller and larger parts with great accuracy [17]. Other electromagnetic simulation software are available for the analysis of large structures [18]: CST (integral equation or asympsotic solver) [19], Ansys HFSS (PO Solver as part of the integral equation solver) [20], and FEKO (MoM/PO hybrid method) [21]. These tools focus primarily on accurately predicting the measured results and are addressed to the antenna development teams. Despite major advances in accelerated computations, they still require a PO integral per beam across the coverage region. When it comes to satcom payload design and system engineering, trade-offs in the accuracy of the antenna patterns can be accepted on the basis of significant acceleration in the computation of the entire multi-beam coverage.

In this paper, we present HERAS (Heriot-Watt reflector antenna solver), a tool for educational and research purposes that comes from the need for a flexible tool for the computation of far-field characteristics of a reflector antenna. HERAS was designed and developed to provide adaptability to system engineering requirements. HERAS is also of interest to antenna engineers working on the design of reflector antenna systems and was, in fact, originally developed to enable the analysis of antenna systems using reflecting polarisers [9,22,23,24,25] at a time when this functionality was not readily available in commercial tools. A description of the tool followed by some projects where the tool has been of contribution are presented in the paper.

## 2. Numerical Techniques for the Analysis of Reflector Antennas

As discussed previously, there are several options commercially available for the analysis of reflector antennas that employ different numerical methods. Finite integration technique (FIT), finite element method (FEM), and method of moments (MoM) are widely used by antenna engineers to solve electromagnetic problems for electrically small structures [26,27,28]. They are generally referred to as “full-wave” or “exact” numerical techniques as they solve Maxwell’s equations, thus providing highly accurate results when convergence is achieved. However, these methods lead to matrices that are of increasing sizes for larger radiating structures (such as reflector antennas), and the computational times and/or the required memory eventually become unmanageable. Other methods have been proposed over the years to reduce the computational requirements when solving this kind of structure, which are based on geometrical optics (GO) and generally referred to as “asymptotic” numerical techniques because of the associated approximations. GO describes the propagation of waves by rays, which is a valid approximation in the case of electrically large structures. However, this ray tracing approximation is not well suited to evaluate interference and diffraction phenomena, as is the case when computing the far-field of an antenna. To this end, hybrid numerical techniques, which combine GO and full-wave techniques, have been developed and are referred to as physical optics (PO). In particular, two methods have received some attention for the analysis of reflector antennas: the aperture and the current distribution methods.

The aperture distribution method obtains the radiated far-field of the reflector antenna from an aperture field distribution derived from ray tracing [29,30]:

(1) $$\vec{E}=jk\frac{e^{-jkr}}{4\pi r}\hat{r}\times {\;\int \int \;}_{S_{a}}-2\hat{z}\times {\vec{E}}_{a}e^{jk{\vec{r}}_{a}\cdot {\hat{a}}_{r}}dS_{a}$$

where $\vec{E}$ is the far-field vector, *k* is the wave number, *r* is the radial component, ${\hat{a}}_{r}$ the unit vector for the far-field direction, and the double integral is evaluated over the aperture plane $S_{a}$ on the aperture field ${\vec{E}}_{a}$ with the integration vector ${\vec{r}}_{a}$. The aperture plane $S_{a}$ is commonly defined as the projected aperture of the reflector surface *S* onto the focal plane, i.e. the plane orthogonal to $\hat{z}$, defining the focal axis, and containing the focal point of the parabola. Essentially, this method applies ray tracing from the feed to the reflector and up to an assumed flat surface, which is then considered to be the radiating aperture. The main advantage of this method is that the integral can be computed with discrete Fourier transform (DFT) which allows the use of algorithms, such as fast Fourier transform (FFT), that significantly reduce the computational cost [31]. In addition, the evaluation can be performed independently of the feed position and illumination pattern. Nevertheless, this method can apply a false symmetry in some cases, which leads to beam squint and side lobe errors [30,32].

**Figure 1:**
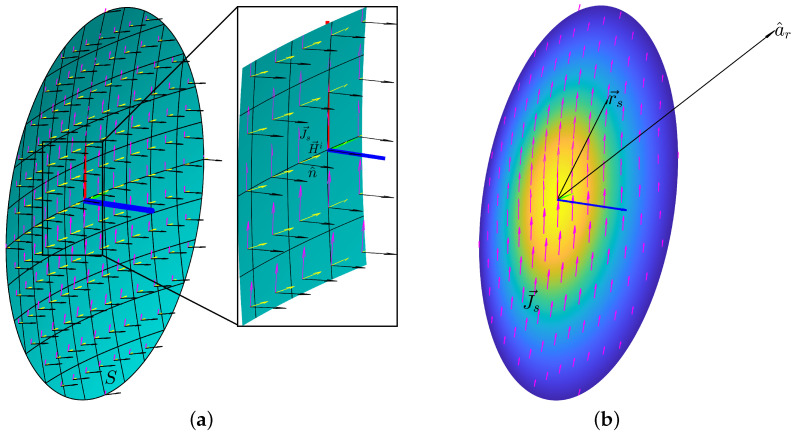
Definition of parameters for PO integral and surface current calculation in a front-fed reflector configuration illuminated by a linearly polarised feed. (**a**) Surface vectors. (**b**) PO integral parameters.

The current distribution method is well-established as the preferred approach to model the radiation characteristics from reflector antennas [15]. Similar to the aperture distribution method, ray tracing is applied in order to obtain the currents on the reflector. For a perfectly conducting surface, the currents can be estimated following the next equation:

(2) $${\vec{J}}_{s}=2\hat{n}\times {\vec{H}}^{i}$$

where ${\vec{J}}_{s}$ is the linear current density, $\hat{n}$ the unit vector, normal to the reflector surface *S*, and ${\vec{H}}^{i}$ the incident magnetic field. It differs from the aperture distribution method in that the far-field is evaluated directly from the integration of these currents:

(3) $$\vec{E}=-j\omega \mu \frac{e^{-jkr}}{4\pi r}{\;\int \int \;}_{S}\left[{\vec{J}}_{s}-\left({\vec{J}}_{s}\cdot {\hat{a}}_{r}\right){\hat{a}}_{r}\right]e^{jk{\vec{r}}_{s}\cdot {\hat{a}}_{r}}dS$$

where $\vec{E}$ is the far-field vector, ω is the angular frequency, μ is the permeability, ${\hat{a}}_{r}$ is the unit vector for the far-field direction and ${\vec{r}}_{s}$ is the integration vector over the reflector surface *S*. These parameters are depicted in Figure 1. While this method gives more accurate results, the numerical implementation of the integral along the reflector surface, which is typically curved, leads to a higher computational cost.

Other techniques to improve the accuracy of the results and the computational costs can be found in the literature [33,34,35]. Diffraction methods can be added to GO and PO in order to more accurately estimate the scattering properties of the surface discontinuities. These are defined in the geometrical theory of diffraction (GTD) [36] and the physical theory of diffraction (PTD) [37]. A modified theory of physical optics (MTPO) was presented in [38] to overcome the shortcomings of PO and approach a solution closer to PTD under some assumptions, such as a perfectly conducting body. However, this methodology has some inaccuracies for short distances as stated in [39]. Multilevel physical optics (MLPO) implements an algorithm based on the hierarchical decomposition of the radiating apertures and the aggregation of their smaller contributions to the PO integral to compute the far-field pattern with a reduction of the computational complexity to a level comparable to the fast Fourier transform [40,41,42]. The iterative physical optics (IPO) technique has been studied for the analysis of open-ended cavities based on the recursive application of the PO integral in order to take into account the multiple reflections inside the cavity [43,44,45]. Several additional numerical techniques and tools with the purpose of speeding up the analysis and synthesis of reflector antennas can be found in the literature [46,47,48,49].

The tool described in this paper uses PO for the analysis of reflector antennas’ radiation characteristics. This technique is generally well adapted to evaluate the performance of electrically large reflectors as generally used in communication satellites. Numerical techniques for the analysis of feeds and their illumination properties in addition to the use of coordinate system transformations are used in the tool and further detailed in [50].

## 3. Description of HERAS

HERAS is a tool developed in Matlab [51] employing object-oriented programming (OOP) for the analysis of reflector antennas using PO theory. It originated from the need to analyse satellite antenna systems using polarising reflective surfaces at a time when this functionality was not readily available in commercial software. Specifically, there was an interest in analysing SFPB antenna systems combining gridded sub-reflectors and linearly polarised feeds [9], as well as cylindro-parabolic reflectors fed by linearly polarised quasi-optical beam-formers [22]. For that purpose, a reflector antenna analysis tool using PO theory was developed in which the perfect electric conductor (PEC) surface was substituted by periodic reflective surfaces. CST was used externally to compute a database of S-parameters of the unit-cell under fully periodic conditions and plane wave incidence, for all needed angles of incidence. Then the field from the feed incident on the unit-cell was combined with the S-parameters to obtain the reflected field. From the reflected field, equivalent currents were computed on the reflector surface, and then the current distribution method, as described in the previous section, was used to compute the far-field. The tool was validated with the design of a flat polarising reflective surface with a non-uniform unit cell layout optimised to reduce cross-polarisation [24]. Good agreement with measurements was achieved, as also reported by other authors employing similar methods [52]. The tool was also validated in the case of a cylindro-parabolic reflector fed by a quasi-optical beamformer [25] and its evaluation is on-going for the design of SPFB antenna systems with gridded sub-reflectors [53].

**Figure 2:**
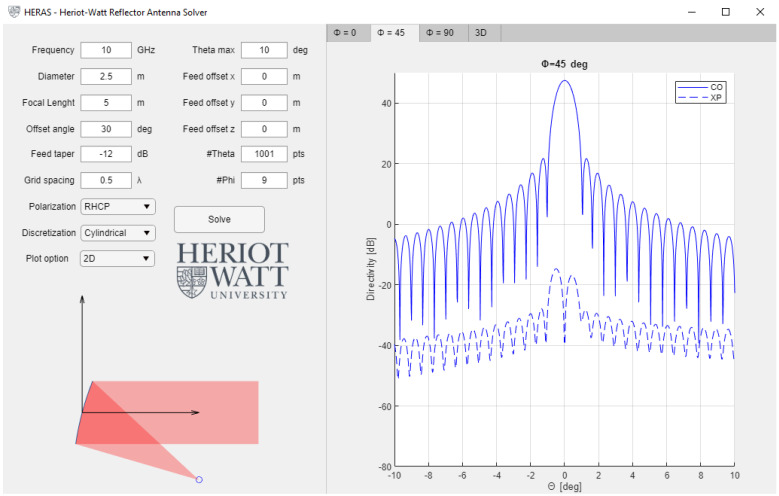
Graphical user interface of HERAS.

HERAS is a simplified version of this internal tool which focuses on reflector antennas with PEC surfaces. While these systems could be analysed using existing commercial software, an open-source code provides more flexibility in adapting the tool to specific needs and in particular system-level MBD analyses, where accuracy may be traded-off with computational efficiency. This proved particularly useful for the analysis of VHTS reflector antenna systems, as mentioned in the introduction. A graphical user interface (GUI) is available which allows us to define a specific reflector configuration and to obtain the performance in the main far-field cuts, as shown in Figure 2. The main parameters of the architecture can be selected in the left side of the window along with the simulation parameters. Next, the “Solve” button can be selected and the results are reported at the right side of the window, including the radiation patterns across the principle planes as well as in a 3D representation. While the GUI can be helpful for quick and easy access to the HERAS solver, a significant advantage of this tool is the access to the source code in order to fully exploit its capabilities for each specific problem.

**Figure 3:**
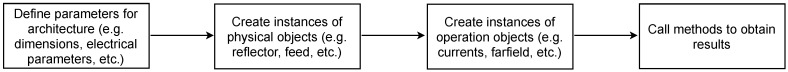
Flowchart to create a script which simulates a reflector antenna configuration.

A folder can be found which contains all OOP classes that can be used in the software. These include classes for objects such as reflector architectures, feeds, etc. This folder can be added to the MATLAB path in order to create individual instances of objects and use them in a specific project. As an example, the flowchart of the steps to create a single MATLAB script to compute the far-field of a reflector antenna configuration using the tool is shown in Figure 3. This script is included along with the tool as an example.

**Figure 4:**
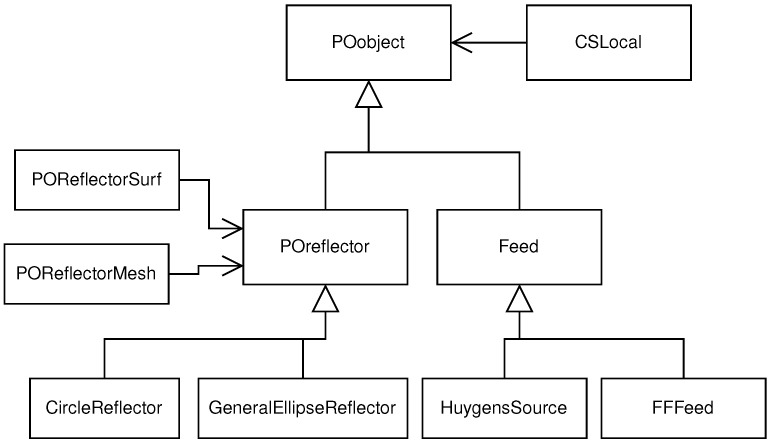
UML diagram of POobject class.

**Figure 5:**
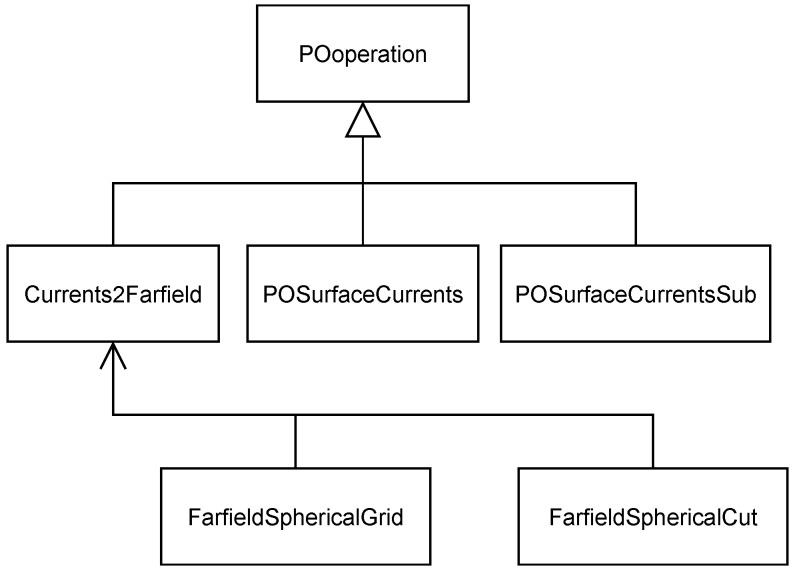
UML diagram for POoperation class.

The basic building blocks of the tool are the classes **POobject** and **POoperation**. The first one is used to define the different objects which compose the reflector antenna architecture such as the feeds and the reflector. The second class is used for the operations related with PO theory and the output of the results. The subclasses **POreflector** and **Feed** inherit from **POobject** (see Figure 4), which define reflector architecture objects and have their own derived classes in order to specify the kind of reflector/feed that is used in the system. **POreflector** also has two associated classes (**POReflectorSurf** and **POReflectorMesh**) to define its geometry, which is used for the PO integral. Every object of the system has its own local coordinate system which is defined with the **CSLocal** class. Once the antenna system is defined with the appropriate objects, PO instructions can be executed, with the use of the **POOperation** subclasses (see Figure 5). **POSurfaceCurrents** and **POSurfaceCurrentsSub** are used for the illumination of the reflector(s) object(s) from the feed(s). The PO integral is computed through the **Currents2Farfield** object, which also stores the results in the format given by **FarfieldSphericalGrid** or **FarfieldSphericalCut**. Each class can perform different operations, which are implemented in the specific class methods. These can include coordinate system conversion, feed illumination, PO integral, plot to figure, etc. The specific operation of each class is detailed in the code.

**Figure 6:**
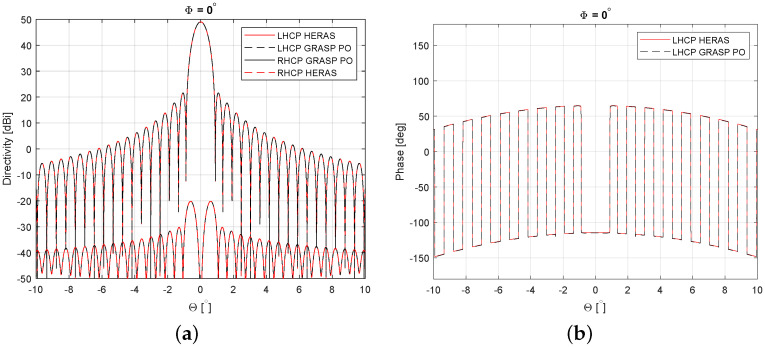
Comparison of the results between GRASP (PO) and Matlab tool in terms of the far-field pattern both in amplitude (**a**) and phase (**b**).

As an example of the performance of the tool, a comparison of the results given by the tool and those obtained with GRASP student version using PO are provided in Figure 6 for a reflector antenna system with an aperture of 100λ at 30 GHz fed by a Gaussian beam in centre-fed configuration.

HERAS can include a spherical wave expansion (SWE) module to import real feeds. As an example, this allows the inclusion of simulated feed far-fields from a full-wave simulation software as well as measured far-field patterns to be used as feeds (**FFFeed** classes in Figure 4) in the reflector system that can be analysed by HERAS [54,55].

## 4. Applications of the Tool

In the previous section, the basic operation of the tool has been described, which allows the simulation of reflector antenna systems using PO theory. As discussed, a core motivation for making the tool available is to provide access to the source code, which allows one to tailor it to the specific needs of a given use case. For example, the computation of the PO integral can be modified to reduce the computational cost at the expense of a reduced yet acceptable accuracy. In this section, some examples of investigations where this tool has been utilised are described.

### 4.1. Efficient Estimation of Antenna System Performance for VHTS Applications in a SFPB Scenario

In a first example, we use HERAS for the analysis of passive (e.g., SFPB/MFPB) antenna architectures. The degrees of freedom in the design of these systems, such as the definition of the optics and the feed array characteristics, call for multidisciplinary optimisations in which the antenna parameters need to be modified multiple times to meet mission requirements. In reflector-based configurations, this is translated to thousands of time-consuming numerical integrations. This is often the bottleneck in the system optimisation time. In this case, computational time is a primary driver provided the coverage metrics are evaluated with an acceptable level of accuracy. Some acceleration techniques applied to the tool described in this paper were presented in [56] in the case of an SFPB configuration with thousands of feeds.

While the beams typically have high directivity in the main lobe, the gain envelope decreases rapidly at the side lobes. Consequently, only a limited area around the main beam is relevant for C/I computation. Since the PO integral is numerically computed for every far-field direction defined in the coverage grid, the computational time is highly dependent on the number of far-field points to be computed. The above observations form the basis of one of the acceleration techniques (in the remaining, referred to as the *threshold* method) which can be implemented directly in HERAS. Instead of computing the far-field pattern for every beam at every coverage point, the computation starts at the estimated centre of the beam and continues with surrounding coverage points until the ratio of the computed field to the peak directivity is below a given threshold. This threshold enables the user to find a suitable compromise between computation time and accuracy. In order to avoid stopping at a null of the far-field pattern, a number of surrounding points are checked in every iteration.

Another acceleration method exploits *interpolation* between beams across the coverage as means to reducing the number of beams numerically evaluated using PO. In particular, this technique exploits the fact that, with the exception of a shift in the pointing direction along the angular domain, neighbouring feeds otherwise produce very similar radiation patterns. The interpolation process is thus performed using two or more surrounding beams to estimate the far-field pattern of a given unevaluated beam. Using the corresponding local coordinates of each beam allows to account for the shift in the angular domain observed between successive beams. The estimated centre point of the beam is therefore computed as the average of the far-field strength obtained in the centre point of the surrounding beams, and the process is repeated for every point, considering the relative distance from the beam centre. This can be performed with one command by virtually aligning the centre of the beams and obtaining the average of their far-fields’ strength. An interpolation path specifies which beams in the coverage are computed with PO and which are interpolated. Since the computational time associated with taking a weighted average to interpolate a beam is negligible compared to the time required to perform the PO integral, the acceleration obtained with this method is directly related to the percentage of beams that are interpolated.

Further computational savings can be achieved by adjusting the number of points defining the reflector mesh grid. This grid defines the discrete set of points along which the surface currents on the reflector, ${\vec{J}}_{s}$, are estimated. This set of points is used to apply the associated PO numerical integration to obtain the far-field, and their number is highly correlated to the computational effort and the accuracy of the results. A coarser mesh reduces the computational time, typically at the expense of a higher error in the side lobe level of the far-field pattern and on the cross-polarisation results. HERAS can be used for a convergence analysis to identify the optimum mesh in terms of aforementioned trade-off. It is noted that HERAS implements parallel computing in order to simultaneously calculate multiple beams using the different cores available in a microprocessor. For this purpose, the Matlab instruction *parfor* has been used [51]. This allows an acceleration factor directly proportional to the number of cores.

**Figure 7:**
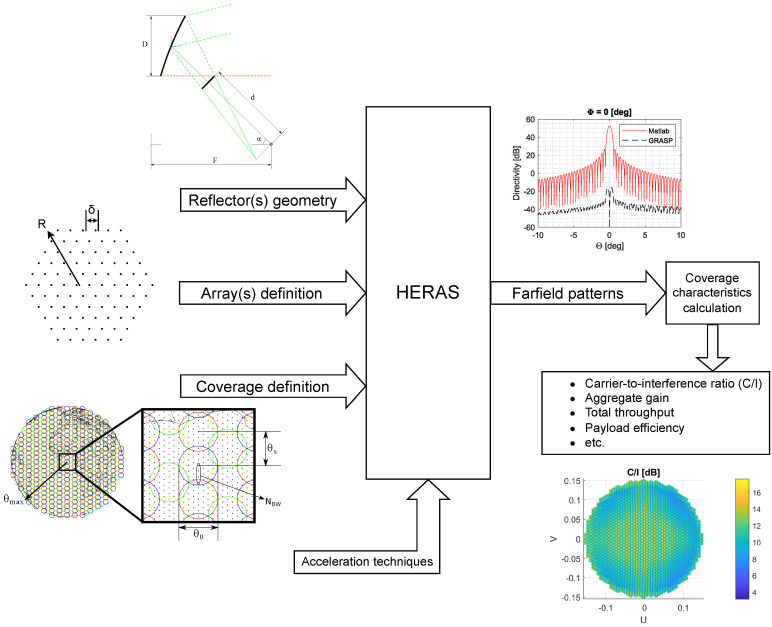
System representation of the Matlab code used to efficiently obtain an estimation of coverage characteristics for a global GEO satellite mission.

A schematic representation of the general working operation of the tool embedding HERAS for the evaluation of reflector antenna systems is shown in Figure 7. The system receives inputs which define the reflector and feeding system, the coverage definition, and the acceleration techniques to be used. Far-field patterns in two orthogonal polarisations are provided by the tool, which are used to compute the C/I values for every point of the coverage. While SFPB configurations commonly employ several reflectors to avoid overlapping of the feeds, the simulation only defines one of them and the feeds are placed in the relative position to their corresponding reflector. This approach simplifies the implementation without affecting the numerical results.

**Figure 8:**
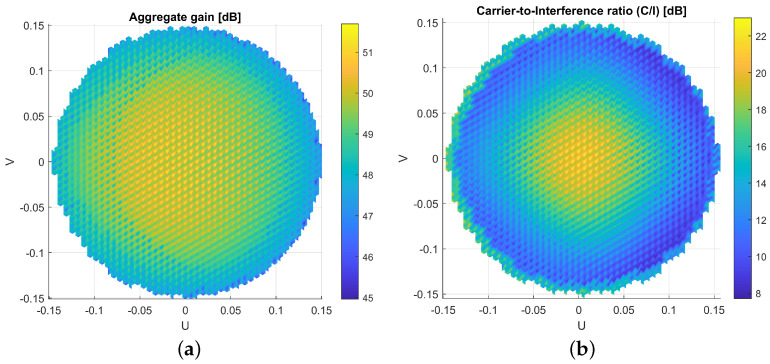
Estimated coverage characteristics in an SFPB scenario. (**a**) Aggregate gain. (**b**) C/I.

**Figure 9:**
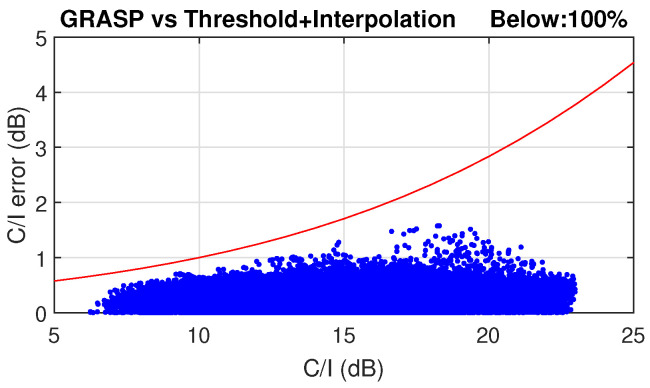
Numerical error resulting from the use of threshold and beam interpolation techniques in an SPFB scenario. An acceleration factor in time of 80 is achieved.

A combination of all these techniques demonstrated a total acceleration factor of 80 in the scenario proposed in [56], which can reduce the computational time from hours to a few minutes with an acceptable accuracy. The antenna design used for this VHTS scenario with a SFPB configuration is also described. Several coverage characteristics can be estimated with the far-field patterns obtained with HERAS. These include C/I, aggregate gain, total throughput, payload efficiency, etc. As an example, C/I and aggregate gain for the SFPB scenario presented in [56] are reported in Figure 8. In addition, Figure 9 shows the evaluated error in a SPFB configuration in terms of C/I over the full Earth coverage when applying the acceleration techniques previously described with reference to the C/I computed using GRASP for all feeds. This error was intended to remain below a certain acceptable level (marked by the red line) enabling a good statistical estimation of the system performance adequate for trade-off analyses driven by worst case values. Far-field points with lower C/I need to be more accurately estimated than those with higher values. The definition of this acceptable error is described in [56].

Taking advantage of the flexibility provided by this approach, a system study may start with a broader parametric evaluation tolerating a slightly higher error, and gradually increase the accuracy as the antenna geometry becomes consolidated. A more refined analysis and fine-tuning of the design may then be conducted at a sub-system level using dedicated tools, such as GRASP, to further improve the performance using a more realistic feed model or introducing reflector surface shaping, for example. It is also noted that the aforementioned acceleration techniques may be implemented using commercial software packages. This is the case for the beam interpolation, for example, which can be directly applied using commercial solvers; the threshold technique is more readily implemented directly in the code and thus benefits from the access to the source code.

### 4.2. Efficient Estimation of an Array Fed Reflector Antenna with a Large Number of Feeds

The fixed coverage characteristics of passive SFPB/MFPB antenna architectures do not allow a favourable match of the satellite capacity to evolving traffic demands. With the technology developments in solid-state power amplification (SSPA) and digital payloads, phased-array active antenna solutions emerge as an attractive candidate to deliver the desired flexibility. Despite the favourable characteristics, the deployment of DRAs at an aperture size suitable to meet stringent link budget requirements for broadband geostationary satellite missions will remain challenging for the foreseeable future. In particular, the limited digital processing capacity of existing and near-future payloads suggests a requirement for extensive implementation of analogue beam-forming networks, which combined with the thermo-mechanical demands associated with distributed SSPA increases the complexity and costs of DRA-based payloads.

AFRs in imaging configuration where the feed array assembly is collectively used to generate each beam are gaining attention for future VHTS systems due to their flexibility [11,57,58]. In these systems, a beam-forming network is required, and precoding and resource allocation can be added in order to increase the overall system performance [12,14,59]. Preferably, these will be implemented in the digital domain, providing a fully reconfigurable software-defined payload such as the recently launched Eutelsat Quantum [60] and other planned broadband missions such as Space Inspire of Thales Alenia Space [61] and OneSat of Airbus [62]. This trend calls for a payload design process aided by multidisciplinary optimisation. Considering the increasing number of beams and the size of the feed arrays, an efficient tool to estimate the multi-beam coverage characteristics is again becoming of significant relevance.

The analysis method for AFR systems entails multiple steps. These include the calculation of the beam-forming coefficients, which defines the weighted contribution of each feed in the formation of each beam; power normalisation, which ensures that each feed-radiated power is within the capability available at the corresponding amplifier; and PO computation, which transforms the reflector illumination to the associated far-field characteristics.

Depending on the ratio between the number of feeds and the number of beams, beam-forming may be considered either at primary or secondary level; in other words, before or after the PO computation. If beam-forming is applied at a primary level, the PO integral is performed for each beam using the equivalent surface currents obtained when the reflector is illuminated with the array field, i.e., the weighted sum of the feed array fields contributing to a particular beam. Primary-level beam-forming could be computationally favourable in cases where the number of beams is lower than the number of feeds, since it requires one PO computation per beam. The acceleration techniques discussed above in the SFPB scenario (e.g., *threshold* and *interpolation*) can be directly applied. It is noted that in this case, the beam-forming matrix should be defined in advance of the coverage computation. In particular, Equation (4) defines the beam-forming computation applied to the currents at the reflector surface of every feed, ${\vec{J}}_{f}$, with the elements excitation matrix, *W*, to obtain the weighted currents for each beam, ${\vec{J}}_{b}$, to which the PO integral is applied to derive the far-field.

(4) $${\vec{J}}_{b}=W{\vec{J}}_{f}$$

**Figure 10:**
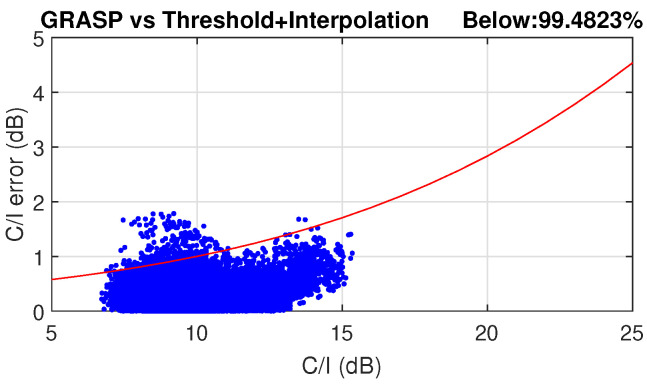
Numerical error resulting from the use of threshold and beam interpolation techniques in an AFR scenario. An acceleration factor in time of 60 is achieved.

The numerical error resulting from the use of the acceleration techniques is shown in Figure 10. An AFR with a reflector of 3 m aperture in offset configuration fed by an array with a magnification factor of 1.6 is used, leading to a similar peak gain of the SFPB configuration discussed in the previous sub-section. The same coverage definition of [56] is applied, so a direct comparison with the SFPB scenario can be obtained. This AFR architecture has higher side lobes, which are primarily contributed in this imaging configuration by the taper at feed array level. Hence, the threshold applied needs to be higher to reduce the error, and consequently the acceleration factor is slightly reduced. Almost 99.5% of points meet the requirements on the numerical error in terms of C/I, which is statistically acceptable.

When beam-forming is performed at a secondary level, the far-field of each defocused feed is computed independently by PO integration and the beam pattern is obtained as a weighted sum of the far-field patterns associated with individual feeds. This leads to a different problem which is described by Equation (5), where ${\vec{E}}_{b}$ represents the beams far-field, ${\vec{E}}_{f}$ the feeds far-field and *W* is the feeds excitation matrix describing the beam-forming.

(5) $${\vec{E}}_{b}=W{\vec{E}}_{f}$$

One advantage of beam-forming at secondary level is that, for a given antenna architecture, the far-field of each feed needs to be computed only once. Different beam-forming strategies and/or precoding processing can then be retrospectively applied to benchmark the antenna performance for different operating scenarios. However, in this case some of the acceleration techniques described previously cannot be directly used. In particular, the threshold method is generally not applicable to the far-field of each defocused feed. This is due to the very broad patterns of the beams associated with highly defocused feeds. Moreover, accurate information on the phase associated with the far-field response of each feed is essential in order to apply beam-forming and obtain the beam patterns. Hence, it is necessary to apply interpolation not only on the amplitude (which in general is sufficient in system studies involving passive SFPB/MFPB antennas) but also on the phase. This requirement, in turn, brings challenges associated to interpolating phase wrapped far-field characteristics. Consequently, exploiting interpolation becomes more challenging.

**Figure 11:**
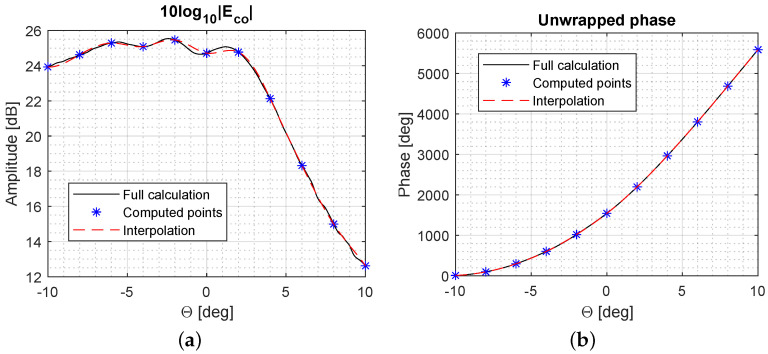
Interpolation of far-field grid points with a subset of them in amplitude (**a**) and phase (**b**). This results have been obtained using the antenna architecture described in [64].

Therefore, a new acceleration method has been studied which, rather than interpolating the far-field characteristics across different beams, exploits interpolation to reduce the number of far-field points that are computed within a given beam. Assuming that the amplitude and phase of the radiation at a subset of far-field points is known, the remaining points are estimated using interpolation performed in both the amplitude and phase domains. An example is illustrated in Figure 11. This method takes advantage of the low spatial variation of the far-field characteristics of defocused feeds and can lead to high acceleration factors, since the computational time highly depends on the number of far-field points to be computed using PO. Of particular significance in this case is the unwrapping of the phase in order to have a smooth variation that can be easily interpolated.

This solution was introduced in [63] and proved useful for parametric studies using different beam-forming techniques and linear precoding in different multi-beam scenarios. A comparative study of different techniques to compute the elements excitation *W* was presented in [64]. The study is focused on direct evaluation techniques in order to provide a rapid estimation of the weights and reduce the computational cost. These include spherical wave fronts from imaging position, spherical wave fronts with gradient, conjugate field-matching (CFM) [65], and zero forcing for beam-forming (ZF) [66].

**Figure 12:**
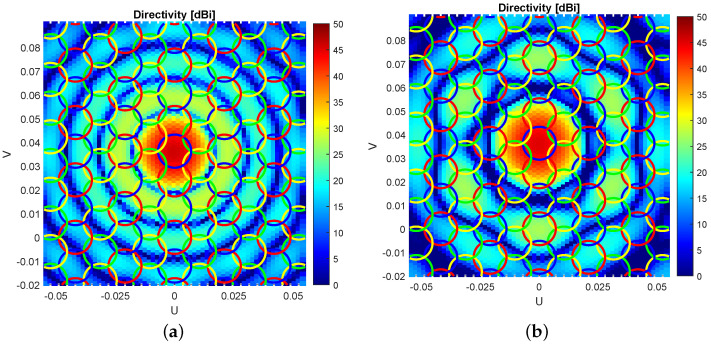
Beam far-field in a four frequency reuse scheme using (**a**) CFM and (**b**) ZF techniques.

CFM and ZF methods can only be evaluated directly if the far-field of each feed is first computed, i.e., when beam-forming at a secondary level is applied in the calculation. These methods produce better system performance in terms of C/I, particularly for amplitude-tapered arrays. In addition, ZF highly reduces interference between beams with respect to CFM as illustrated in Figure 12. A four frequency reuse scheme is implemented and ZF is applied to the estimated centre of the beams with the same frequency. It can be seen that the side lobe levels on cells with the same colour are reduced in Figure 12b.

## 5. Conclusions

The HERAS software for the analysis of reflector antennas using physical optics has been presented. This tool, with source code in MATLAB, is available freely for educational and research purposes and may be of interest to antenna engineers for parametric studies and custom reflector antenna system design. HERAS may also be of interest to satellite communicationspayload and system engineers due to its adaptability and applicability in end-to-end performance investigations. Some projects where the tool has been of contribution related to VHTS systems have been presented, showing the potential of the tool for the design, analysis, and optimisation of such systems, which employ thousands of beams to achieve higher capacities. Specifically, this tool has been used for the efficient estimation of SFPB antenna systems, where several acceleration techniques were introduced in order to reduce the computational cost of the calculation of thousands of beams. In addition, the tool has also demonstrated its potential for the analysis of AFR configurations in combination with several signal processing techniques such as beam-forming and precoding for the analysis of these systems in terms of the final system performance.

## Data Availability

The source code of HERAS and data reported in this paper are available from the corresponding author, upon reasonable request.
